# Molecular epidemiology of Rotavirus causing diarrhea among children less than five years of age visiting national level children hospitals, Nepal

**DOI:** 10.1186/s12887-017-0858-0

**Published:** 2017-04-07

**Authors:** Subhash Dhital, Jeevan Bahadur Sherchand, Bharat Mani Pokhrel, Keshab Parajuli, Niranjan Shah, Shyam Kumar Mishra, Sangita Sharma, Hari Prasad Kattel, Sundar Khadka, Sulochana Khatiwada, Narayan Parajuli, Basistha Rijal

**Affiliations:** 1 National Public Health Laboratory, HIV Reference Unit, Kathmandu, Nepal; 2 Department of Microbiology, Tribhuvan University Teaching Hospital, Kathmandu, Nepal; 3 Department of Microbiology, Universal Medical College, Bhairahawa, Nepal; 4 Department of Microbiology, ManMohan Memorial Institute of Health Sciences, Kathmandu, Nepal

**Keywords:** Rotavirus, Diarrhea, Nepal

## Abstract

**Background:**

Rotaviruses are the major cause of diarrhea among the infants and young children all over the world causing over 500,000 deaths and 2.4 million hospitalizations each year. In Nepal Rotavirus infection positivity rates ranges from 17.0 to 39.0% among children less than 5 years. However, little is known about the molecular genotypes of Rotavirus prevailing. The objective of this study was to estimate the burden of Rotavirus gastroenteritis and determine the genotypes of Rotavirus among children less than 5 years.

**Methods:**

The cross sectional study was conducted from January to November 2014 among children less than 5 years old visiting Kanti Children’s Hospital and Tribhuvan University Teaching Hospital.

Rotavirus antigen detection was performed by Enzyme Linked Immunosorbent Assay (ELISA) using ProSpecT Rotavirus Microplate Assay. Among the Rotavirus antigen positive samples, 59 samples were used for Rotavirus RNA extraction. Multiplex PCR was performed to identify G type comprising G1-G4, G8-G10 and G12 and P type comprising P[4], P[6], P[8], P[9], P[10], and P[11].

**Results:**

A total of 717 diarrheal stool samples were collected from patients ranging from 10 days to 59 months of age. Rotavirus antigen positive was found among (*N* = 164)22.9% of patients. The highest number of the diarrhea was seen in January. Molecular analysis of Rotavirus genotypes revealed that the predominant G-Type was G12 (36%) followed by G9 (31%), G1 (21%), G2 (8.6%). The predominant P- type was P6 (32.8%) followed by P8 (31%), P10 (14.8%), P4 (14.8%). A total of seven G/P type combinations were identified the most common being G12P [6] (35.8%), G1P [8] (15.1%), G9P [8] (15.1%).

**Conclusion:**

Rotavirus diarrhea is, mostly affecting children from 7 to 24 months in Nepal, mostly occurring in winter. The circulating genotypes in the country are found to be primarily unusual genotypes and predominance of G12P[6]. It is recommended to conduct genotyping of Rotavirus on large samples before starting vaccination in the country.

**Electronic supplementary material:**

The online version of this article (doi:10.1186/s12887-017-0858-0) contains supplementary material, which is available to authorized users.

## Background

Rotaviruses are non-enveloped, double stranded RNA viruses with 11 gene segments under Reoviridae family having seven major groups (A-G) [1]. Group A Rotaviruses are the major causes of diarrhea among the infants and young children all over the world [2] causing over 500,000 deaths and 2.4 million hospitalizations each year [3]. The virus is composed of three concentric layers; the outermost shell contains VP7 or G protein and VP4 or P protein, which are able to produce neutralizing antibodies during infection of a host [1]. Such neutralizing antibodies are the basis of dual classification of group A rotaviruses into G and P types. With the advent of molecular techniques (Reverse transcriptase, RT-PCR), classification into G and P type on the basis of genes encoding the G and P proteins has become widely accepted standard technique [4].

Virtually every child around the globe experiences Rotavirus diarrhea by the age of 3–5 years [2]. The higher standards of living style, good hygiene and proper sanitation followed in developed countries are not merely sufficient to prevent Rotavirus diarrhea. Although the incidence of diarrhea is similar in developed and developing countries, the Rotavirus diarrhea differs characteristically between them, in developing countries the severity as well as mortality is very high, many strains occur at a time and disease occurs at early age [5]. In addition, in developing countries diarrhea results in a deadly cycle of malnourishment and diarrhea [6].

With no availability of antivirals, vaccines are hoped to prevent disease, reduce morbidity and mortality of the disease. World Health Organization (WHO) has recommended the inclusion of Rotavirus vaccination of infants into all national immunization programs [7]. Eighty four countries including 36 low income countries have introduced Rotavirus vaccination and global coverage is estimated to be 23%. Although the efficacy of oral vaccines in developing countries, in presence of other enteric infections, was dubious; recent data shows vaccination was successful in substantially reducing severe diarrhea and/or rotavirus disease [8]. However, Rotavirus vaccination has not been included in national immunization program in Nepal. And studies on Rotavirus infection from 1999 to 2007 showed positivity rates ranging from 17.0 to 39.0% among children less than 5 years [9].

Globally, G1, G2, G3, and G4 constitute more than 88.5% of globally identified strains [5]. At odds with global scenario, the most frequently reported G types included G12, G1, G2, G9 from Nepal [10–13]. The first description of G12 rotaviruses dates back to the 1990s from Philippines which were characterized serologically and by nucleotide sequencing as G12P[4]. Beginning in 2002, reports of the detection and increased prevalence of G12 strains have appeared from Asia (Thailand, India, Korea, Japan, Bangladesh, Nepal, and Saudi Arabia) and the America (the United States, Argentina, and Brazil) [14].

There are regional and geographical differences as well as difference over time in same region in strain distribution [7]. The enormous diversity of Rotavirus is mainly because of point mutations, genetic reassortment or introduction of animal viral strains to human beings [15]. Therefore, rotavirus surveillance is needed to monitor the prevalence and possible changes of the different G and P types circulating in the region.

The study was conducted to estimate the burden of Rotavirus gastroenteritis as well as genotype of rotavirus among children less than 5 years of age visiting national level children hospitals, Kanti Children’s Hospital and Tribhuvan University Teaching Hospital.

## Methods

The cross sectional study was conducted from January to November 2014 among children less than 5 years of age visiting national level children hospitals, Kanti Children’s Hospital and Tribhuvan University Teaching Hospital. These both hospitals are situated in Kathmandu city, capital of Nepal. These are among the largest hospitals of the country. Kanti Children’s Hospital is only central/national pediatric hospital of the country.

Seven hundred seventeen single diarrheal stool specimens were collected from children with acute diarrhea who were referred to the observation ward and who were admitted to the hospital for not more than 3 days. The 10–20% suspensions of the specimens were made using phosphate buffered saline for antigen testing and genotyping in different vials and stored at −20 °C.

Stool samples were initially tested for group A Rotavirus antigen using ProSpecT Rotavirus enzyme immune assay (Oxoid Ltd., Basingstoke Hants, UK) and subsequently random 59 Rotavirus antigen positive samples were selected for RNA extraction using the commercial QIAamp Viral RNA Mini Kit (QIAGEN Inc). The tests were performed as specified by manufacturer.

### RT PCR

The extracted dsRNA was converted to cDNA as described by Gouvea et al. [16]. The formed cDNA was used for subsequent PCR reactions.

### Typing by multiplex PCR

#### G–Typing

The first round of G type PCR was targeted to gene segment 9. Amplification of the fragment was performed with primers, VP7-F (5’ATG TAT GGT ATT GAA TAT ACC AC3′) and VP7-R (5’AAC TTG CCA CCA TTT TTT 3′) in a 50 μl of a PCR mixture (5 U Taq DNA polymerase; 10 mM each dNTP’s; 50 mM MgCl_2_; 20 pmol of each primer; 10X buffer). The second round, a multiplex PCR was designed to identify the genotypes G1-G4, G8-G10 and G12 [12, 17].

#### P-Typing

The first round of P type PCR was targeted to gene segment 4. Amplification of fragment was performed with primers, VP4-F (5’TAT GCT CCA GTN AAT TGG3’) and VP4-R (5’ATT GCA TTT CTT TCC ATA ATG 3’) in a 50 μl of a PCR mixture (5 U Taq DNA polymerase; 10 mM each dNTP’s; 50 mM MgCl_2_; 20 pmol of each primer; 10X buffer) The second round, a multiplex PCR was designed to identify the genotypes P[4], P[6], P[8], P[9], P[10], and P[11] [18].

## Results

Seven hundred and seventeen patients less than 5 years of age were enrolled in the study with age from 10 days to 59 months. The highest number of patients (*N* = 341) belonged to age group 0–12 months followed by (*N* = 202) 13–24 months. In total there were 475 (66.2%) males and 242(33.8%) females. The dataset generated during the study are available in the data_diarrhea repository within the Additional file 1.

### Occurrence of Rotavirus diarrhea

All samples were examined for Rotavirus using ELISA. The ELISA of Rotavirus antigen was found to be positive among 22.9% (*N* = 164) of the isolates.

### Monthly occurrence of Rotavirus diarrhea

**Fig. 1:**
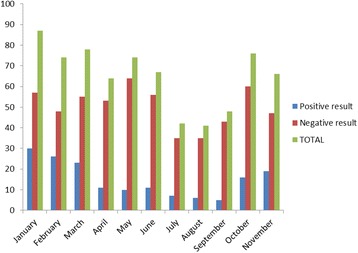
Monthly occurrence of Rotavirus diarrhea

The highest number of rotaviral diarrhea (*N* = 30) was seen in January followed by (*N* = 26) in February as shown in Fig. 1.

### Age wise distribution of Rotavirus ELISA result

The highest number of Rotavirus positive diarrheal patients was detected on 0–12 month age group category which accounted 56.0% of total Rotavirus positive samples as shown in Table 1.

**Table 1:** Age wise distribution of Rotavirus ELISA result

Age group (months)	Positive Result	Negative Result	Total
0–12	92 (56.1%)	249	341
13–24	45(27.5%)	157	202
25–36	14(8.5%)	69	83
37–48	8(4.8%)	43	51
49–60	5(3.05%)	35	40

#### Distribution of G-Type of Rotavirus

Molecular analysis of Rotavirus genotypes revealed that the predominant G-Type was G12 (*n* = 21, 36%) followed by G9 (*n* = 18, 31%), G1 (*n* = 11, 21%), G2 (*n* = 5, 8.6%). Out of five G-types, G3 (*n* = 4) was least found genotype representing 3.4%, while 1.8% was found to be untypable.

**Fig. 2:**
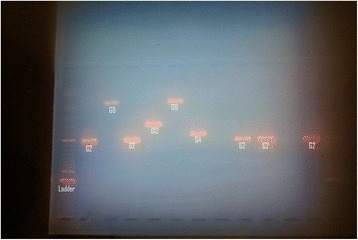
Gel run of G-type PCR showing Ladder (Lane 1), G2(Lane2), G9(Lane3), G2(Lane4), G12(Lane5), G9(Lane6), G4(Lane7), UT(Lane8), G2(Lane09), G2(Lane10), Negative control(Lane 11), G2(Lane 12) from left to right.

The genotype was determined according to the electrophoresis of the product which differed according to the basis base pairs as shown in Fig. 2.

#### Distribution of P-Type of Rotavirus

Molecular analysis of Rotavirus genotypes revealed that the predominant P-type was P[6] (*n* = 19, 32.8%) followed by P[8] (*n* = 17,31%), P[10] (*n* = 1014.8%), P[4] (*n* = 9, 14.8%). Similarly, 6.6% of isolates were found to be untypable.

#### Genotypic distribution of Rotavirus

In total seven G/P type combinations were identified, including G12P[6] (35.8%), G1P[8] (15.1%), G9P[8] (15.1%) which was the most commonly detected Rotavirus strain type, followed by G9P[4] (9.4%), G2P[4] (7.5%). (Table 2).

**Table 2:** Genotypic distribution of Rotavirus

Distribution of genotypes		
S.N	Genotype	Total
1.	G1P[8]	8
2.	G1P[10]	2
3.	G2P[4]	4
4.	G2P[10]	1
5.	G3P[10]	2
6.	G9P[4]	5
7.	G9P[8]	8
8.	G9P[10]	2
9.	G12P[6]	19
10.	G12P[8]	1
11.	G12P[10]	1
12.	MIXED	4
13.	PARTIAL	5

## Discussion

The Rotavirus antigen positive was found among 22.9% of the isolates in the study similar to other studies in Nepal; 25.9% by Ansari [19] et al., 17% by Uchida R [20] et al. and 33% by Sherchand et al. [13]. The incidence rate is comparable to the reports from India, Malaysia, Japan and Taiwan (20–25%) but much lower than Thailand, Myanmar and Vietnam (38.1–56%) [21]. These higher prevalence countries also relied on similar method of detection i.e. Enzyme immunoassay of Rotavirus antigen, the higher incidence rate in these countries is ascribed to low occurrence of other etiology (bacteria) of diarrhea, increased level of economic standards, proper water supply and good hygiene.

In the study overriding Rotavirus infections was observed in 1 year’s age group accounting 56.0% of total Rotavirus positive samples (*P* value =0.03,<0.5). In the group, most infections (34.2% of total) occurred at the age of 7 to 12 months. And more than 80% of Rotavirus infections are found within the 2 years of age. Similarly, various studies have found that most of Rotavirus infections occur within 2 years of age, particularly after 7 months of age [19, 22–24]. Thus, vaccination at an early age can be beneficial to prevent majority of cases of diseases.

Researches have shown that infant below 4 months of age were initially protected to some extent by maternal antibodies against severe rotavirus diarrhea, and breast feeding as another independent factor preventing the diarrhea, which can be a reason to have peak after 7 months [25, 26].

Rotavirus diarrhea is a disease of winter, recognized as a winter gastroenteritis and vomiting but seasonality may vary year to year [27]; in this study, peak in the number of RVGE cases was observed between January and March and marked the seasonal variation, with peaks occurring in cooler and drier months of the year. However, seasonal nature of Rotavirus is not universal, and in countries within 10° of the equator, infection occurred year round. Similar type of seasonal variation has been reported by Uchida R et al. [20], Sherchand et al. [13], Sherchan et al. [28] from studies done in Nepal. Notably, in this study, the cases were found whole year round; in contrast Uchida et al. [20] found no cases in August and September and Sherchan [28] et al. did not find any case of Rotavirus diarrhea in July.

Molecular analysis of Rotavirus genotypes revealed that the predominant G type was G12 (36%) followed by G9 (31%), G1 (21%) and G2 (8.6%). The predominance of G 12 is in agreement with other studies as well, in a 3 year study by Sherchand et al. from November 2005 to October 2008 in Nepal, G12 was predominant in all 3 years, 2005–2006 (50%), 2006–2007(29%), and 2007–2008 (33.7%) [29]. In comparison to previous studies in Nepal, there are subtle changes in the genotypes. In a study by Ansari et al. (2013), G 12 (56%) was most predominant followed by G2, G1 and G9 [19]. However, in a study by Uchida et al. (2006) G1 was predominant, followed by G 12 and G2. In current scenario, G12 is being regularly reported in high frequencies from South East Asian countries-India, Bangladesh with considerable diversity suggesting Ganges region can be its origin from where it might have transmitted across the globe [30–32]. Remarkably, we have reported 31% of G9, higher percentage in comparison to previous studies in Nepal. G9 was first reported from Philadelphia and after 1990s it has been found with increased frequency worldwide, it has been reported from India, Brazil, Italy, the United States, Bangladesh, Malawi, the United Kingdom, France, and Australia [33, 34]. When the molecular study of Rotavirus begun from 2003 in Nepal by Uchida et al., in that first study G9 was not found, in subsequent studies by Sherchand et al. and Ansari et al. there were G9 genotypes occurrence comprising 2–6% [19, 20, 28]. Alike kind of increase in G9 has also been observed in neighboring country India, from 2 to 10% in 2003–2007 to around 40% in 2013 [35]. The increment of G9 can be because of the escape recognition by immune system of Nepalese children where G 12 was predominant in previous years. In spite of all, emerging unusual G12 genotype continues to be a predominant in our context and circulating genotypes are within the cluster of G12, G9, G1, G2 and G3 [11–13, 19, 20, 28].

In this study the major P-type was P[6] (32.8%) followed by P[8] (31%), P[10] (14.8%) and P[4] (14.8%), previous studies also concordantly depicted either P[6] or P[8] to be the leading one [11–13, 19, 20, 28]. P[6] has been commonly detected from symptomatic infections as major genotype nowadays, although previously, P[6] VP4 genotypes were detected from asymptomatic infections only [36–38]. In the study P[10] was found among less than one fourth of isolates in association with G1, G2, G3, G9 and G10, although P[10] was not reported before. The P-type (P[10]) as a unusual serotype has also been reported from African countries like Cameroon, Ghana, Libya [39].

The most common genotype combination in this study is found to be G12P[6] (36%) followed by G1P[8] (15.1%), G9P[8] (15.1%) G9P[4] (9.4%), G2P[4] (7.5%). Comparing the Rotavirus genotype result from Nepal in various studies, G12P[6] was found to be 12% in 2006 [20], 29% in 2011 [28], 46.4% in 2013 [19] and 36% in this study showing prevailing characteristics of G12 P[6]. Although globally 5 G-P combinations (G1P[8], G2P[4], G3P[8], G4P[8]) and G9P[8]) is responsible for approximately 90% of all human rotavirus infections; the unusual serotypes of Rotavirus infections in our context is anticipated. It has been described that unusual G and P combinations constitute more than 14% of reported rotavirus isolates from Asia, 27% from Africa, 11% from South America, 5% from North America, 1.4% from Europe, and 0.1% from Australia [40]. Unlike developed countries where one or two genotypes predominate in a season [41], large number of genotype combinations along with unusual genotypes are comprehended in our study. Mixed Rotavirus infection was observed in 7.5%. G 1/G 12, P[4]/P[6] and P[4]/P[8] are the pattern of mixed infections in this study.

The percentage of G1 P[8] was decreasing constantly from 70% in 2006 [20] to 19% in 2011 [40] and 2.2% in 2013 [19]. Epidemiological and molecular studies in many countries show complex patterns of change from 1 year to the next of Rotavirus serotypes associated with diarrhea from the same geographical areas [42].

The Rotavirus vaccination has not been incorporated in the national immunization schedule in Nepal. However, there are instances of immunization on the advice of clinician. Because of high burden of Rotavirus gastroenteritis the necessity of vaccination can be appreciated. The steep decrease in burden of diarrhea after implementation of vaccination program can be expected from the experience of countries like USA, Finland, Belgium, Brazil, Venezuela, Mexico where Rotavirus vaccination has been adopted. The reduction of all cause diarrhea hospitalizations is larger in less than 1 year old (35.6%) compared to one to 4 year old children (12.3%) as noted in the study by Gurgel et al. [43].

## Conclusion

Rotavirus diarrhea is, mostly affecting children from 7 to 24 months in Nepal, mostly occurring in winter. The circulating genotypes in the country are found to be primarily unusual genotypes and predominance of G12P[6]. It is recommended to conduct genotyping of Rotavirus on large samples before starting vaccination in the country.

## Additional file

Additional file 1: The data generated during the study. It includes the data generated like age/sex, month of hospital visit, number of diarrhea and vomiting in excel sheet. (XLSX 50 kb)

## Abbreviations

μl: Microliter
cDNA: Complementary DNA
DNA: Deoxyribonucleic Aicd
dNTP’s: Deoxynucleotide triphosphate
dsRNA: Double stranded RNA
ELISA: Enzyme Linked Immuno Sorbent Assay
IRB: Institutional Review board
MgCl_2_: Magnesium Chloride
mM: Milli mole
PCR: Polymerase Chain Reaction
RNA: Ribonucleic Acid
RT-PCR: Reverse Transcriptase Polymerase Chain Reaction
RV: RotaVirus
TUTH: Tribhuvan University Teaching Hospital
VP: Viral Protein
WHO: World Health Organization
